# DISH vs Spondyloarthritides

**DOI:** 10.31138/mjr.31.1.81

**Published:** 2020-03-31

**Authors:** Fotini Angelopoulou, Pantelis Kraniotis, Dimitrios Daoussis

**Affiliations:** 1 University of Patras Medical School, Patras, Greece,; 2 Departments of Radiology and Rheumatology,; 3 Patras University Hospital, University of Patras Medical School, Patras, Greece

**Keywords:** axial spondyloarthritides, diffuse idiopathic skeletal hyperostosis

**Figure 1.**
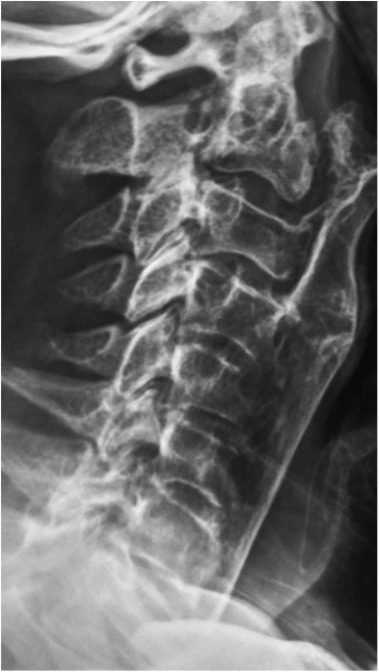
Lateral C-spine radiograph. There is extensive bulky calcification of the anterior longitudinal ligament (ALL) from C2 level extending to the upper thoracic spine, affecting more than four contiguous vertebrae. The lesion impinges upon the upper airways. There is no evidence of calcification of the posterior longitudinal ligament. The facet joints and disk spaces are preserved.

**Figure 2.**
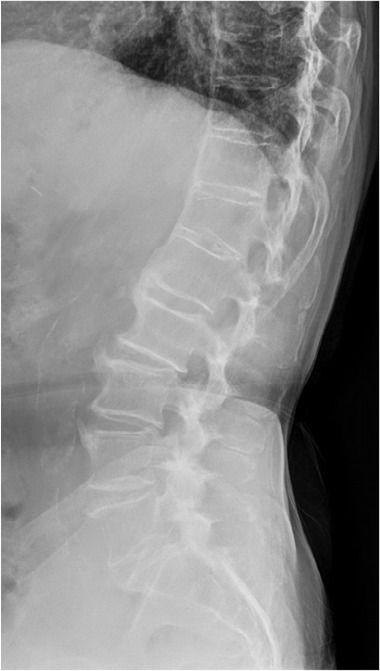
Lateral lumbar radiograph. There is bony bridging of the lower thoracic spine and T12-L1 bodies anteriorly. Bulky anterior syndesmophytes are depicted in the lumbar vertebrae form L1 to L4. However, there is no evident bridging except at L1–L2 level.

**Figure 3.**
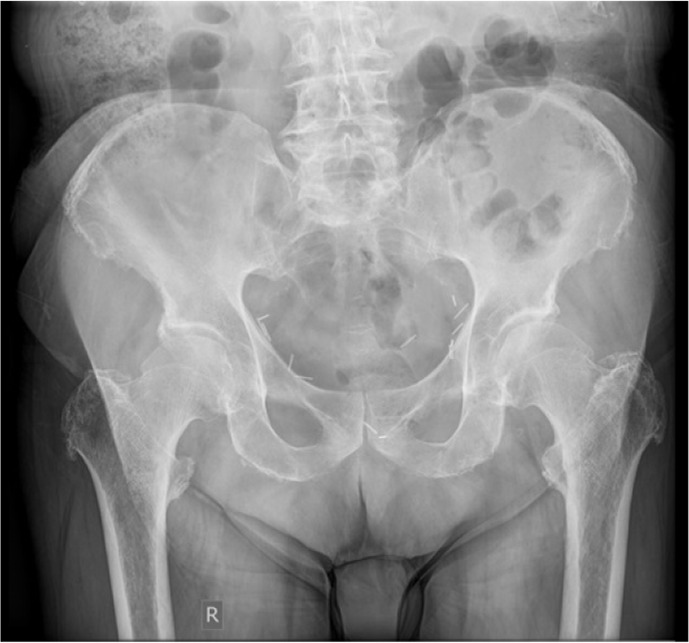
Pelvis AP radiograph. There is whiskering enthesopathy more pronounced in the greater and lesser trochanters and more subtle in the iliac crests and the ischial tuberosities. The sacroiliac joints are fused to some extent and sclerotic.

An 85-year-old man presented to the Rheumatology clinic with symptoms consistent with an episode of acute gouty arthritis. During physical examination it was also found that the patient had severe dorsal kyphosis and marked limitation of cervical spinal mobility. His medical history was unremarkable. There was no history of inflammatory back pain or stiffness. He only reported mild neck pain without functional impairment and had no need for analgesics. There was also no history of psoriasis, inflammatory bowel disease, uveitis or dactylitis; family history was also negative. The patient underwent a series of radiological investigations that showed pronounced bulky calcification of the anterior longitudinal ligament extending from his cervical to upper thoracic spine along with bony bridging of the lower thoracic spine and bulky syndesmophytes in the lumbar vertebrae (*Figure 1*, *Figure 2*). X-ray of his pelvis (*Figure 3*) showed whiskering enthesopathy, involving the greater and lesser trochanters and to a lesser extend the iliac crests and ischial tuberosities. All these radiologic findings pointed towards diffuse idiopathic skeletal hyperostosis (DISH). However, his pelvic radiograph showed that both sacroiliac joints were fused, something that is atypical for DISH. Presence of sacroiliac joint (SIJ) fusion or ankyloses may pose a diagnostic dilemma in distinguishing DISH from radiographic axial spondyloarthritis (SpA). We should bear in mind, however, that these clinical entities may coexist in rare cases.^1^ DISH is a pathological entity characterized by calcification and ossification of soft tissues, mainly ligaments and entheses. It involves, but is not limited to the axial skeleton, mostly the thoracic spine and peripheral entheses.

Periarticular hyperostosis of the hands, knees, elbows and quadriceps tendon insertion are also a common feature of DISH.^1^ It has always been considered a primarily radiographic entity with minor clinical significance compared to other spinal diseases. It manifests clinically as limitation of spinal mobility, kyphosis and rarely dorsolumbar pain. In rare cases it may also cause dysphagia, hoarseness and stridor, none of which was experienced by our patient despite the extensive bone overgrowth in the cervical region close to the upper airway.

DISH and SpA are two entirely separate pathological entities that only share the involvement of the axial skeleton and peripheral enthesis. DISH affects predominantly middle-aged and elderly people, and has a strong association with diabetes mellitus and obesity.^2^ Ankylosing spondylitis (AS), on the other hand, is an inflammatory disorder of the axial skeleton that typically develops in early adulthood. Its main symptoms include inflammatory back pain and stiffness and decreasing range of motion of the spine. Prominent radiographic features include bilateral and symmetric sacroiliitis, vertebral body squaring and paramarginal syndesmophytes that run parallel to the spine giving a bamboo-like appearance. Main differences between SpA and DISH are presented in *Table 1*. Differentiating between DISH and SpA is not always a straightforward task. It is considered that the main difference between DISH and AS is the lack of SIJ erosions, sclerosis and bony fusion in DISH. However, a 2017 study compared individuals who fulfilled the classification criteria for DISH to healthy controls and CT scans of SI joints were evaluated for abnormalities. It was found that anterior and posterior bridging, entheseal bridging and joint fusion of the SIJ was detected more significantly in patients with DISH compared to controls raising serious concerns regarding the use of SIJ abnormalities as a “absolute” criterion to discriminate between DISH and SpA.^3^

**Table 1.** Main differences between SpA and DISH.

	SpA	DISH
Clinical Characteristics	Adolescents, young adults Hx of psoriasis, IBD, uveitis, dactylitis Family history Hx of back pain before the age of 45 Inflammatory back pain or stiffness Postural abnormalities Response to NSAIDs	Elderly, but can be found in patients under 45 yo Usually asymptomatic Limitation of spinal mobility Kyphosis, rarely dorsolumbar pain dysphagia, hoarseness and stridor insome cases strong association with DM andobesity
Laboratory	High CRP levels HLA-B27	Metabolic syndrome associated features
Radiographic findings	Primarily affects the anulus fibrosus of the intervertebral discs Continuous spine lesions Shiny corner sign Bamboo spine Vertebral body squaring Syndesmophytes Sacroiliitis: Joint space stenosis, subchondral sclerosis and ankylosis of the lower one third (synovial) part of the SI joint	Continuous bulky calcification of the anterior longitudinal ligament Osteophytic bridging of at least 4 continuous vertebrae, typically of the thoracic spine Whiskering enthesopathy of the greater and lesser trochanters, ischial tuberosities and iliac crests Periarticular hyperostosis of the hands, knees, elbows and quadriceps tendon insertion

The majority of patients with DISH do not have spinal symptoms, and the disease is usually diagnosed based on X-rays performed for other reasons. In clinical practice, when evaluating X-rays with continuous vertebral bony bridging, it is advisable to also assess pelvic X-rays for evidence of sacroiliitis. In asymptomatic patients with no evidence of sacroiliitis, a diagnosis of DISH can be safely made. In our case, the X-ray of the pelvis instead of verifying the diagnosis of DISH, as expected, further complicated the differential diagnosis by raising the suspicion of SpA. However, in this case the clinical scenario was obvious and strongly supportive of DISH. Our patient was an elderly man who never experienced inflammatory spinal pain and never required analgesics. Moreover, the extensive bony overgrowth in the cervical region was strongly suggestive of DISH.

Since our patient was asymptomatic and of advanced age, no further investigation was pursued.
